# Caffeic and Chlorogenic Acids Synergistically Activate Browning Program in Human Adipocytes: Implications of AMPK- and PPAR-Mediated Pathways

**DOI:** 10.3390/ijms21249740

**Published:** 2020-12-21

**Authors:** Liliya V. Vasileva, Martina S. Savova, Kristiana M. Amirova, Zhivka Balcheva-Sivenova, Claudio Ferrante, Giustino Orlando, Martin Wabitsch, Milen I. Georgiev

**Affiliations:** 1 Department of Plant Cell Biotechnology, Center of Plant Systems Biology and Biotechnology, 4000 Plovdiv, Bulgaria; vasileva@cpsbb.eu (L.V.V.); msavova@cpsbb.eu (M.S.S.); amirova@cpsbb.eu (K.M.A.); sivenova_jivka@abv.bg (Z.B.-S.); 2 Laboratory of Metabolomics, The Stephan Angeloff Institute of Microbiology, Bulgarian Academy of Sciences, 4000 Plovdiv, Bulgaria; 3 Department of Pharmacy, G. d’Annunzio University, 66100 Chieti, Italy; cferrante@unich.it (C.F.); giustino.orlando@unich.it (G.O.); 4 Division of Pediatric Endocrinology and Diabetes, Department of Pediatrics and Adolescent Medicine, Ulm University Medical Center, 89077 Ulm, Germany; Martin.Wabitsch@uniklinik-ulm.de

**Keywords:** chlorogenic acid, caffeic acid, adipocytes, obesity, browning, anti-obesity effect, molecular docking

## Abstract

Caffeic acid (CA) and chlorogenic acid (CGA) are phenolic compounds claimed to be responsible for the metabolic effects of coffee and tea consumption. Along with their structural similarities, they share common mechanisms such as activation of the AMP-activated protein kinase (AMPK) signaling. The present study aimed to investigate the anti-obesity potential of CA and CGA as co-treatment in human adipocytes. The molecular interactions of CA and CGA with key adipogenic transcription factors were simulated through an in silico molecular docking approach. The expression levels of white and brown adipocyte markers, as well as genes related to lipid metabolism, were analyzed by real-time quantitative PCR and Western blot analyses. Mechanistically, the CA/CGA combination induced lipolysis, upregulated AMPK and browning gene expression and downregulated peroxisome proliferator-activated receptor γ (PPARγ) at both transcriptional and protein levels. The gene expression profiles of the CA/CGA-co-treated adipocytes strongly resembled brown-like signatures. Major pathways identified included the AMPK- and PPAR-related signaling pathways. Collectively, these findings indicated that CA/CGA co-stimulation exerted a browning-inducing potential superior to that of either compound used alone which merits implementation in obesity management. Further, the obtained data provide additional insights on how CA and CGA modify adipocyte function, differentiation and lipid metabolism.

## 1. Introduction

Obesity and overweight are prevalent health problems that are associated with type 2 diabetes, insulin resistance, cardiovascular complications, low-grade chronic inflammation and, hence, increased risk of cancer development [1]. Considering the limited number of clinically available anti-obesity drugs [2], finding promising candidates for novel drug leads is on urgent demand. Development of effective treatments counteracting obesity is directed to several, indeed, overlapping approaches: (i) obstruction of excessive lipid accumulation and adipocyte hypertrophy [3,4,5,6]; (ii) resolution of the chronic inflammation in white adipose tissue (WAT) of the obese [7,8,9]; (iii) induction of metabolically healthy adipocyte recruitment [10,11,12]; (iv) promotion of energy expenditure through enhanced lipolysis, thermogenesis and browning of the WAT [13,14,15,16,17,18]. 

Adipose tissue is classified as WAT, which is a depot of energy storage, and brown adipose tissue (BAT), mainly responsible for thermogenesis and energy expenditure [16,17]. Classic brown adipocytes are characterized with higher numbers of mitochondria whose inner membrane is rich in uncoupling protein 1 (UCP1) and which are activated upon β3-adrenergic stimulation or cold exposure [15,17,19,20,21]. The energy-dissipating ability of BAT could help to recover the energy imbalances resulting from metabolic disorders such as obesity and type 2 diabetes. Another type of adipocytes—inducible brown-like adipocytes, localized in WAT—was described in recent decades and termed as beige or brown-in-white [14,16,19,22,23]. Cold exposure, β3-adrenergic agonist or prolonged peroxisome proliferator-activated receptor γ (PPARγ) agonist treatment, among other stimuli, trigger the biogenesis of beige adipocytes in WAT depots, also known as browning [6,17,23,24,25]. Like BAT, beige adipocytes expend energy in the form of heat, which has resulted in increased interest in the utilization of browning of white adipocytes as an option for cell-based therapy in obesity-related metabolic disorders [14,18,19,21,26,27]. Furthermore, pharmacological activation of the transition of white into beige adipocytes has been exploited as a promising approach for the drug-development of novel anti-obesity leads [19,22,25,28].

Natural compounds have been investigated as potential browning agents [29]. The AMP-activated protein kinase (AMPK), a key regulator of energy homeostasis, peroxisome proliferator-activated receptor gamma co-activator 1 alpha (PGC-1α), PPARs and the β-adrenergic receptors have been described, among others, as key targets mediating their thermogenic effects [24,28,29,30,31,32,33]. 

Caffeic acid (3,4-dihydroxycinnamic acid, CA) and its derivatives are commonly occurring in plant species, especially as major secondary metabolites in coffee and tea [34,35,36,37]. Chlorogenic acid (CGA) is an ester of caffeic and quinic acids that is long recognized as beneficial in obesity and type 2 diabetes management [31,36,37,38,39,40,41]. Popularly termed as tea polyphenols [35], CA and CGA are known to inhibit lipid oxidation and reactive oxygen species formation in vitro [42,43], in vivo [38,44] and ex vivo [36,45]. Additionally, anti-inflammatory [41,44], anti-hyperglycemic [36,38,39,40,41], hepatoprotective [39,40,46] and cardioprotective [42,44] effects are reported for CGA. Clinically relevant data support the positive correlation between CA and CGA plasma concentrations in healthy women and decreased risk of type 2 diabetes development [47]. Existing literature implied CGA as an inducer for non-shivering thermogenesis, mainly affecting adipogenic differentiation through AMPK activation [29,36,38]. Earlier studies also report that the anti-diabetic and anti-hyperlipidemic effects of CGA are AMPK-dependent [40].

In the current study, we have employed a human Simpson–Golabi–Behmel syndrome (SGBS) preadipocyte cell strain as a model of white adipogenic differentiation in aiming to evaluate the potential browning induction upon co-treatment with caffeic and chlorogenic acids. In silico analysis was performed to predict the affinity of both compounds towards key target proteins. Molecular pathways affected were revealed with gene expression analysis by real-time qPCR (RT-qPCR) and targeted protein detection of adiponectin, CAAT/enhancer-binding protein alpha (C/EBPα) and PPARγ by Western blot analyses. Furthermore, additional insights into the mechanism of action of caffeic and chlorogenic acids in human adipocytes were disclosed.

## 2. Results

### 2.1. Caffeic and Chlorogenic Acids Display Potential to Interact with AMPK- and PPAR-Related Proteins

**Figure 1:**
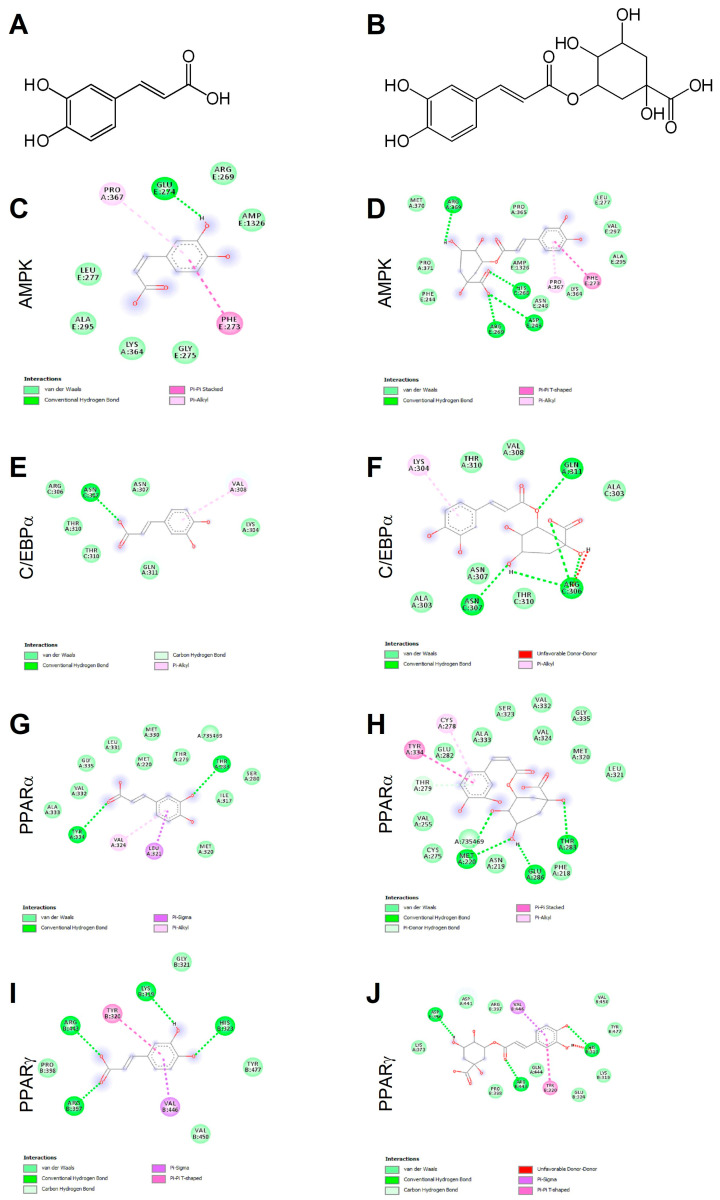
Chemical structures of caffeic and chlorogenic acids and molecular docking models of putative interactions with target proteins. Caffeic acid chemical name: (E)-3-(3,4-dihydroxyphenyl)prop-2-enoic acid; Mw 180.16 g/M (**A**). Chlorogenic acid chemical name: (1S,3R,4R,5R)-3-[(E)-3-(3,4-dihydroxyphenyl)prop-2-enoyl]oxy-1,4,5-trihydroxycyclohexane-1-carboxylic acid; Mw 354.31 g/M (**B**). Putative interaction between caffeic acid and AMP-activated protein kinase (AMPK; PDB: 4CFF); Free energy of binding (ΔG) and affinity (Ki) are −6.4 kcal/M and 20.6 µM, respectively (**C**). Chlorogenic acid and AMPK (PDB: 4CFF); ΔG −8.1 kcal/M; Ki 1.2 µM (**D**). Caffeic acid and CAAT/enhancer-binding protein alpha (C/EBPα; PDB: 1NWQ); ΔG −4.7 kcal/M; Ki 362.5 µM (**E**). Chlorogenic acid and C/EBPα (PDB: 1NWQ); ΔG −6.1 kcal/M; Ki 34.2 µM (**F**). Caffeic acid and peroxisome proliferator-activated receptor α (PPARα; PDB: 2P54); ΔG −6.0 kcal/M; Ki 40.5 µM (**G**). Chlorogenic acid and PPARα (PDB: 2P54); ΔG −7.6 kcal/M; Ki 2.7 µM (**H**). Caffeic acid and peroxisome proliferator-activated receptor γ (PPARγ) (PDB: 2P4Y); ΔG −5.8 kcal/M; Ki 56.8 µM (**I**). Chlorogenic acid and PPARγ (PDB: 2P4Y); ΔG −7.4 kcal/M; Ki 3.8 µM (**J**).

Ligand binding mechanisms of small molecules could be predicted with high reliability through molecular docking analysis [45,48]. Chlorogenic acid being a caffeic acid derivative shares similar bioactivities, mainly mediated through AMPK activation, and is reported to promote browning in WAT [29,40]. In order to deeply investigate the molecular interactions between CA (Figure 1A) and CGA (Figure 1B) with AMPK, C/EBPα and PPARα and -γ, a docking study was conducted. The related binding affinities, measured as binding free energy (∆G) and affinity constant (Ki), and the non-covalent interactions were evaluated. The orientation of the docked compounds is presented in Figure 1C–J. Chlorogenic acid displayed higher affinities than CA towards all target proteins which could be due, albeit only partially, to the number of hydrogen bonds and pi-interactions predicted for CGA by the virtual screening software. In this regard, the capability of CGA to modulate the activity of both PPARα (Figure 1H; Ki 2.7 µM) and PPARγ (Figure 1J; Ki 3.7 µM), previously reported by others [38,39], agrees with the predicted Ki values in the low micromolar range, whereas the low affinities (Ki ranging from 40.5 to 56.8 µM) displayed by CA towards PPARα (Figure 1G) and PPARγ (Figure 1I) scale back the importance of this latter phenolic compound as a PPAR modulator. These findings are consistent, at least in part, with an in vivo study [38] that demonstrated the capability of CA and CGA to stimulate PPARα activity, evaluated as hepatic protein expression in obese mice. Our docking study also supports the previously reported activation of AMPK induced in hepatocytes upon CA and CGA stimulation [39,40]. Specifically, the higher binding affinity displayed by CGA (Figure 1D, Ki 1.2 µM) compared to CA (Figure 1C, Ki 20.6 µM) further strengthens the anti-obesity effects of CGA [41]. By contrast, both tested compounds showed very low affinities towards C/EBPα (Figure 1E,F; Ki ranging from 34.2 to 362.5 µM), thus suggesting a direct pharmacological interaction with this protein target as ambiguous.

The obtained data underlined the importance of AMPK and PPARα/-γ as targets for CA and CGA bioactivity and added further details on their specific molecular interactions.

### 2.2. Caffeic and Chlorogenic Acids Co-Treatment Promotes Lipolysis in Human Adipocytes

Human preadipocyte cell strain derived from an infant patient with the rare Simpson–Golabi–Behmel syndrome (SGBS) serves as a valuable tool to study adipogenesis and fat cell metabolism in vitro [3,12,20,49].

Facilitation of glucose metabolism and improvement in insulin sensitivity of fat, liver and muscle tissues, mainly through protein kinase B (AKT) phosphorylation or AMPK activation, has been reported for both CA and CGA [36,38,39,40]. To examine whether their combined anti-obesity potential is superior to their effects as single treatments, we have employed SGBS preadipocytes as an in vitro model system. White adipogenic differentiation was induced in SGBS cells in the presence of increasing concentrations of CA, CGA and the combination of both compounds. 

**Figure 2:**
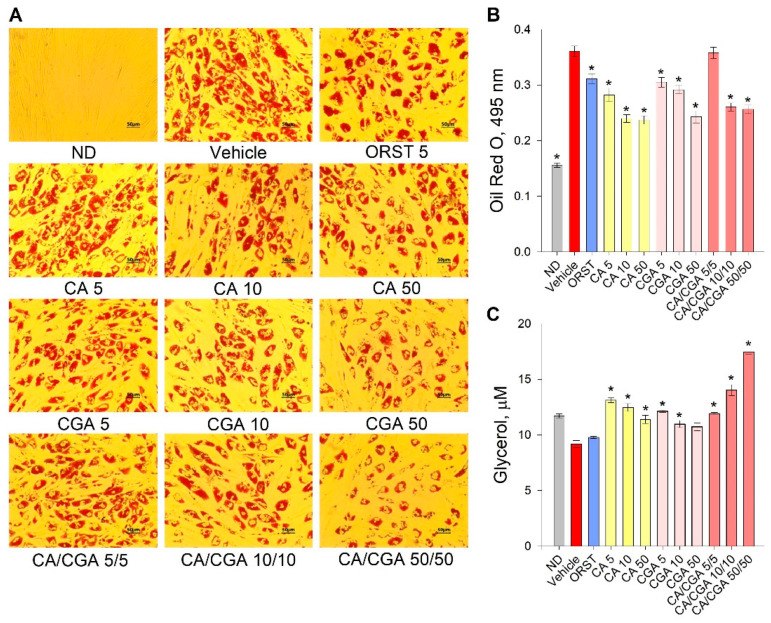
Caffeic and chlorogenic acids decreased lipid accumulation and enhanced glycerol release in human Simpson–Golabi–Behmel syndrome (SGBS) adipocytes. Representative pictures captured with 20× magnification (scale bar 50 μm) after the Oil red O staining (**A**). Absorbance of the Oil red O solution at 495 nm (**B**). Free glycerol concentration (µM) in the cell culture media (**C**). Data are expressed as mean ± SEM; each experimental group consisted of at least six technical replicates from three independent biological experiments. * *p* < 0.05 compared to vehicle control group.

Initial evaluation of the effect of CA and CGA on adipogenesis was performed via the estimation of intracellular lipids with an Oil red O assay. Despite the fact that the orlistat (ORST) mechanism of action is through gastric/pancreatic lipase inhibition and its direct effect on adipocytes is equivocal [50], we have employed it as reference drug as it is approved and widely prescribed in anti-obesity therapeutic regimens [51]. A dose-dependent decrease in lipid accumulation was observed in the cells treated with both phenolic acids (Figure 2A), which was confirmed by the spectrophotometric measurements of the extracted dye (Figure 2B). The co-treatment of CA/CGA did not exceed the anti-adipogenic effect of either of the compounds alone, nor that of the positive control ORST. However, in regard to lipolysis, an additive effect was observed upon CA/CGA co-stimulation (Figure 2C), which is in agreement with previous studies reporting that CGA activates basal lipolysis [38,40]. 

Combined, these findings suggest that CA/CGA treatment inhibits adipogenesis and alters adipocyte lipolysis to an extent exceeding that of their individual application, and such stimulation could be eventually associated with enhancement of intracellular lipids turnover.

### 2.3. Combination of Caffeic and Chlorogenic Acids Activates Browning Gene Expression

White adipogenic differentiation is driven by the activation of C/EBPα and PPARγ in tandem as core transcriptional regulators [1,10]. Subsequently, adipogenesis results in upregulation of sterol regulatory element-binding protein 1 (SREBP1) which controls fatty acid biosynthesis, which, in turn, elevates acetyl-CoA carboxylase (ACC) and fatty acid synthase (FASN). Additionally, adipocyte maturation is characterized by an increase in the expression of adiponectin (ADIPOQ), leptin and fatty acid binding protein 4 (FABP4) [1,2,3,4]. 

Transdifferentiation of white adipocytes towards the brown-like phenotype could be induced via several mechanisms, most of which appeared to be PGC-1α/PPAR-dependent. PGC-1α stimulation induces mitochondrial biogenesis and, as a result, increases UCP1 expression in the mitochondria [17,20,49,50,51]. Additionally, browning promotion in WAT is characterized by upregulation of a specific beige marker such as that of T-box 1, tumor necrosis factor receptor superfamily member 9 (CD137), cell death activator CIDE-A (CIDEA) and pyruvate dehydrogenase kinase isoform 4 (PDK4) [17,19,28].

**Figure 3:**
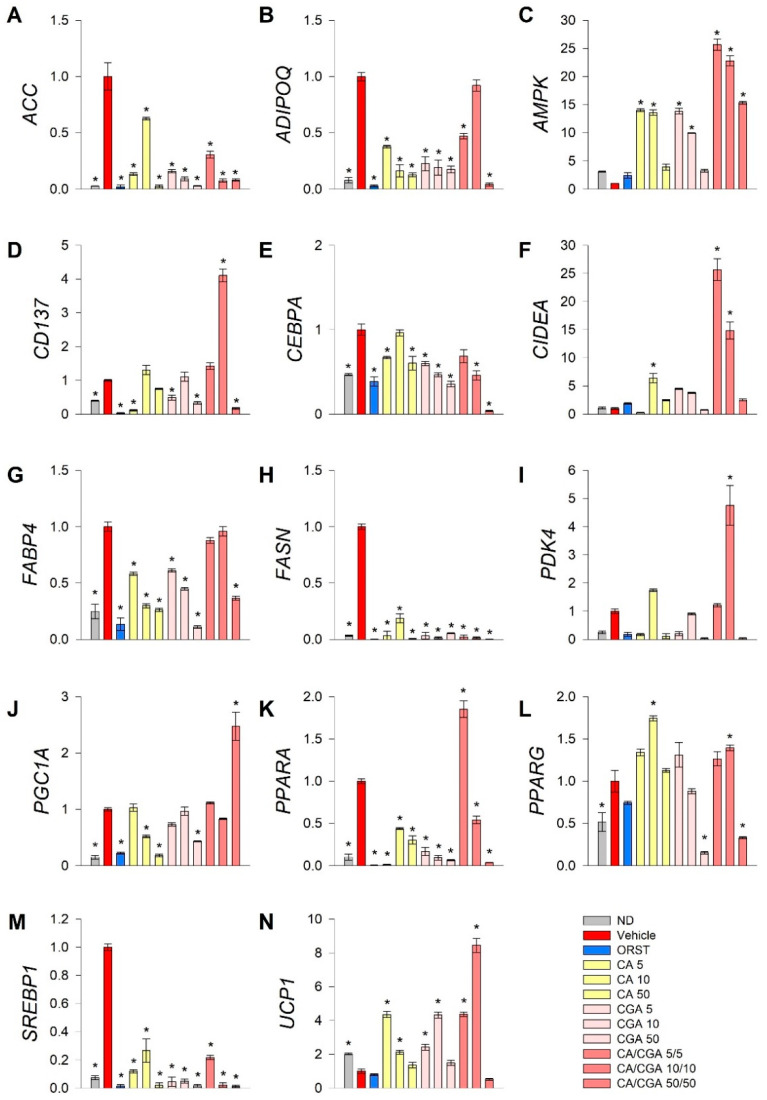
Combined treatment with caffeic and chlorogenic acids altered adipogenic gene expression and induced browning markers’ transcriptional activation in human SGBS adipocytes. Relative mRNA expression (∆∆Cq) normalized to vehicle control group for the following genes: (**A**) acetyl-coA-carboxylase (*ACC*), (**B**) adiponectin (*ADIPOQ*), (**C**) AMP-activated protein kinase (*AMPK*), (**D**) tumor necrosis factor receptor superfamily member 9 (*CD137*), (**E**) CAAT/enhancer-binding protein alpha (*CEBPA*), (**F**) cell death activator CIDE-A (*CIDEA*), (**G**) fatty acid binding protein 4 (*FABP4*), (**H**) fatty acid synthase (*FASN*), (**I**) pyruvate dehydrogenase kinase isoform 4 (*PDK4*), (**J**) peroxisome proliferator-activated receptor gamma co-activator 1 alpha (*PGC1A*), (**K**) peroxisome proliferator-activated receptor alpha (*PPARA*), (**L**) *PPARG*, (**M**) sterol regulatory element-binding protein 1 (*SREBP1*) and (**N**) uncoupling protein 1 (*UCP1*) from the RT-qPCR. *RPL13A* and *TUBB* were applied as reference genes. Each sample was analyzed in triplicate from three independent experiments. Data are presented as mean ± SEM. * *p* < 0.05 compared to the vehicle control group.

To explore further the underlying mechanisms by which CA and CGA modify adipogenesis, RT-qPCR analysis was performed of key genes from fatty acid metabolism (*ACC*, *FASN*, *SREBP1*), transcriptional regulation of white adipogenic differentiation (*ADIPOQ*, *CEBPA*, *FABP4*, *PPARG*) and brown/beige markers (*CD137*, *CIDEA*, *PDK4*, *PGC1A*, *PPARA*, *UCP1*). Additionally, we have examined *AMPK* mRNA expression levels as both CA and CGA are known as AMPK activators (Figure 3). 

Data from the gene expression analysis indicated that the lipogenic markers *ACC* (Figure 3A), *FASN* (Figure 3H) and *SREBP1* (Figure 3M) were significantly downregulated upon treatment with CA and CGA alone or as co-stimulation. The expression levels of *AMPK* (Figure 3C), *CEPBA* (Figure 3E), *PPARA* (Figure 3K) and *PPARG* (Figure 3L) were also influenced, which appeared in agreement with the in silico molecular docking predictions. Interestingly, an additive effect was observed regarding the transcript accumulation of *AMPK* (Figure 3C) and the modulation of *PPARA* (Figure 3K) upon combinatorial treatment with both compounds at the lower concentrations used, which was not detected for the *CEBPA* (Figure 3E) and *PPARG* (Figure 3L) gene expression levels. Moreover, the white adipogenic markers *ADIPOQ* (Figure 3B) and *FABP4* (Figure 3G) were suppressed by CA and CGA alone, but to a lesser extent by their combination, thus suggesting preservation of the adipocyte metabolic functions. 

We also confirmed the previously suggested browning effect of CGA [29,36] in human white SGBS adipocytes through the RT-qPCR data analysis. Although the extent of the browning effect was lower in the case of CA and CGA stimulations alone, the brown adipogenic markers *CD137* (Figure 3D), *CIDEA* (Figure 3F) and *UCP1* (Figure 3N) were significantly increased in all treatment groups. An additional increase was detected upon co-treatment with CA/CGA in SGBS adipocytes for *PDK4* (Figure 3I), *PGC1A* (Figure 3J), *PPARA* (Figure 3K) and *UCP1* (Figure 3N).

Additionally, data from the hierarchical cluster analysis revealed that the gene expression profiles of the SGBS cells exposed to CA/CGA co-treatment at the lower concentrations of 5/5 μM and 10/10 μM distantly differ from the vehicle-treated white adipocytes and strongly resemble that of brown-like adipocytes (Supplementary Materials Figure S2). These findings support the observation of significantly induced lipid evacuation, determined by the glycerol release assay (Figure 2C). However, a further increase in the concentrations of CA and CGA in their combination treatment (50/50 μM) did not result in an enhanced browning of white adipocytes, but rather in potent inhibition of adipogenesis and fatty acid synthesis. Combination of 50 μM of each compound provoked a dramatic decrease in all tested genes except *AMPK* and *PGC1A* (Figure 3).

Collectively, these findings support the conclusion that the AMPK signaling pathway is the most prominent for the anti-adipogenic effects of CA and CGA in adipocytes. Co-treatment with both phenolic acids altered mRNA expression profiles in human white adipocytes towards browning or suppressed lipogenesis.

### 2.4. Caffeic and Chlorogenic Acids Induce Browning in Human Adipocytes via PPAR-Dependent Mechanism

**Figure 4:**
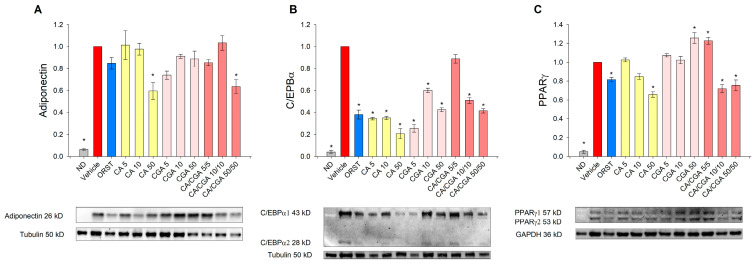
Caffeic and chlorogenic acids diminished adiponectin, CAAT/enhancer-binding protein alpha (C/EBPα) and peroxisome proliferator-activated receptor γ (PPARγ) protein production in human SGBS adipocytes. Western blot analysis was performed to examine the protein level of adiponectin (**A**), C/EBPα (**B**) and PPARγ (**C**). Data are expressed as mean ± SEM and are representative of three independent experiments. * *p* < 0.05 compared to the vehicle control group.

We next sought to determine the adipogenesis-related factors adiponectin, C/EBPα and PPARγ at a protein level by Western blot analysis because the effect of CA and CGA on these proteins in human adipocytes is insufficiently explored. Adiponectin, which serves as a marker for white adipogenic differentiation [3], was decreased upon CA and CA/CGA treatments at the highest concentrations (Figure 4A). Consistent with the results from the mRNA expression analysis (Figure 3E), C/EBPα protein levels were reduced by CA and CGA and to lesser extent from their combination (Figure 4B). Notably, regarding PPARγ activity, both compounds produced opposing effects (Figure 4C). Caffeic acid hampered PPARγ activity in a dose-dependent manner, while CGA produced an agonistic effect. These observations corroborate the data from the in silico analysis (Figure 1I,J). Interestingly, the combination of both CA and CGA at the lower concentrations of 5 μM each significantly activated PPARγ to a level almost equal to the highest concentration of CGA alone (50 μM). However, increase in the doses of the co-treatment inversed the interaction with PPARγ to predominantly antagonistic, represented by the lowered protein levels. 

Accordingly, the data on the mRNA expression profiling and immunoblotting analysis pointed to the presence of an additive anti-adipogenic effect of CA/CGA co-treatment mediated through direct modulations of AMPK and PPARα/-γ as major mechanisms.

## 3. Discussion

Our overall findings disclose previously unappreciated mechanisms of action of CA and CGA in the regulation of adipocyte differentiation and metabolism. Although both compounds share structural similarities and their additive anti-obesity effect has been previously suggested [39,47], differences in gene expression patterns resulting from CA/CGA combinatorial treatment in human adipocytes has not been evaluated. Moreover, our findings expand the understanding of the underlying pathways involved in the CA and CGA potential synergism with relevance to obesity management.

The human SGBS cell strain has been established as a model to study adipocyte biology and metabolism [3,12,20]. Among the advantages of SGBS preadipocytes is their capability of adipogenic differentiation in vitro that is retained for over 50 generations, as well as their superior relevance to human biology over the commonly used 3T3-L1 murine preadipocytes [12]. However, when compared to other human preadipocyte cell lines, there are also some concerns about the SGBS cells’ changeful phenotype [52,53,54]. Several reports present evidence that after the 14th day of differentiation and up to the 28th day, SGBS cells transiently overexpress UCP1, PPARγ and other genes when compared to PAZ6 brown adipocyte cells and primary human adipocytes from non-obese [52] or obese people [53]. Such results would bring into question their use as a model of browning as their response to thermogenic stimulation could differ from that of primary human white adipocytes. However, this transient phenotype could be implemented in studies aiming at elucidation of the mechanisms of spontaneous unstimulated white-to-brown adipocyte transition [54]. In the present study, we have focused our research interest on clarification of the potential synergistic effect of CA and CGA in human SGBS preadipocytes subjected to a white adipogenic differentiation for 9 days following a standard well-described protocol [3,12,40]. The vehicle-treated control adipocytes on day 9 of differentiation had a gene expression profile characteristic for white adipocytes, revealed by the RT-qPCR analysis. Therefore, we consider that all differences between the control adipocytes and the experimental treatment groups are representing the effect of CA, CGA or their combination.

Anti-diabetic and anti-obesity effects of CA and CGA supplementation involved hepatic PPARα activation, increased insulin production and inhibition of key enzymes from the lipid biosynthesis that produced body weight reduction and improved metabolic parameters compared to high-fat diet-induced obese mice [38]. Treatment with high concentrations of CGA 200 μM alone was shown to effectively improve glucose metabolism through upregulation of the mRNA expression of both PPARα and -γ in murine adipocytes [41]. Another study also reported anti-obesity and anti-lipidemic effects related to hepatic AMPK activation in HepG2 and leptin-deficient mice [40]. Improved insulin sensitivity mediated via phosphatidylinositol-3-kinase (PI3K)/AKT was also reported in a HepG2 model of insulin-resistant hepatocytes upon stimulation with phenolic acid fraction rich in CA and CGA [39]. In agreement to that, phenolic compounds derived from coffee silverskin extract, including CA and CGA, prevented insulin resistance and improved lipid metabolism in murine adipocytes model via the insulin/PI3K/AKT pathway and elevated PGC-1α and UCP1 protein expression levels [36]. In addition, a plant-derived extract rich in phenolic compounds (among which CA and CGA were present) exerted anti-inflammatory potential and improved PPARγ activity in lipopolysaccharide-stimulated murine 3T3-L1 adipocytes [43]. 

To date, a variety of natural compounds have been suggested with thermogenic and, hence, browning potential, including CGA [24,28,29,30,32,33,48]. For instance, the anti-obesity effects of polymethoxyselenoflavones [28] and mangiferin [24] involve stimulation of lipolysis through the protein kinase A-mediated pathway and induction of brown-like phenotype of WAT. Resveratrol is also known to promote browning though the AMPK/sirtuin (SIRT)-dependent pathway [30,31,33,48]. However, the activation of AMPK by combinatorial treatment with CA and CGA has not been evaluated in the context of browning in human adipocytes. 

Extensively growing numbers of studies are exploring alternative browning switches among which CIDEA, UCP1 and certain sirtuins are present [15,21,22,26,27]. Activation of AMPK in the WAT promotes the generation of beige adipocytes populations with enhanced thermogenic potential independently of UCP1 expression [22,32,33]. Enhanced glucose utilization in muscle tissue, suppressed gluconeogenesis and inhibited fatty acid synthesis via direct ACC modulation in WAT are mediated by AMPK as a central regulator of the cellular energy homeostasis [33,40]. Phosphorylation of AMPK at a cellular level is imperative for activation of AMPK downstream signaling [55]. Both CA and CGA are described with high potential to activate AMPK in various cell types [29,36,38,40], which is supported by our in silico molecular docking analysis. Therefore, we have considered that for the aim of the current study, changes in *AMPK* gene expression level would be sufficient to represent the effect upon CA and CGA co-stimulation in adipocytes and the focus at a protein level should be on specific adipogenic targets. Correspondingly, our data showed that CA/CGA co-treatment at concentrations of 5/5 and 10/10 μM robustly upregulated *AMPK* to levels exceeding those after CA or CGA use alone, resulting in induction of brown phenotype and inhibited lipogenic gene expression (*ACC*, *FANS*, *SREBP1*) in human adipocytes. In addition to the suppressed de novo lipogenesis, the CA/CGA combination induced lipolysis to higher extent than CA or CGA alone, thus further supporting their additive anti-adipogenic effect. Moreover, these results hinted that CA/CGA treatment could promote browning, as intracellular degradation of lipids is known to fuel such transformations in WAT. Fatty acid mobilization though lipolysis also provides ligands for the PPAR target genes which play a central role in WAT remodeling [21,28,56]. 

Stimulation of PPARα or -γ induces browning in white adipocytes though PGC-1α-, UCP1- or SIRT-mediated pathways [22,25,57]. The PGC-1α protein is of key importance for the maintenance of insulin sensitivity and mitochondrial biogenesis in both hepatic and adipose tissues and its activation leads to browning through increase in UCP1 expression [18,24,29,58]. Our data showed increased mRNA expression levels for *PCG1A*, *UCP1*, as well as other browning markers genes such as *CD137*, *CIDEA* and *PDK4* upon co-stimulation with the lower doses of CA/CGA in adipocytes, which clearly indicates its browning effect. The substantial downregulation of all genes except *AMPK* and *PGC1A* that was provoked by the co-treatment of CA/CGA at doses of 50 μM of each could be a result from one or both of the two compounds outreaching its stimulatory effect in adipocytes and reverse it to potent inhibitory action. Another possible explanation is that CA/CGA co-treatment at doses above 50 μM obstructs white adipogenic differentiation at an earlier stage and in a stronger manner than the lower doses, which is reflected in substantial inhibition in adipogenic genes at day 9. 

Repressive PPARγ modulators acting as inverse agonists are suggested as promising therapeutic options in obesity management and are associated with enhancement in bone formation and improved WAT metabolic functions [9,48,59,60]. Furthermore, recent insights into the structural mechanisms of the PPARγ repressive function [48] permit better understanding of the interpretation of the interactions between chemical ligands and PPARγ protein. In this regard, the molecular docking analysis performed in the present study provides relevant data on the CA and CGA potential to directly influence PPARα and -γ activity. The in silico experiments permitted to predict the orientation of these phenolic compounds within the active sites of the aforementioned target proteins. Furthermore, the role of PPARα and -γ proteins in the CA/CGA mechanism of action was confirmed with our data at transcriptional and protein levels. Strikingly, bi-directional agonistic/antagonistic modulation of PPARγ was observed upon co-stimulation with CA and CGA at the transcriptional level. One possible explanation could be derived from the molecular docking data that predicted substantial differences in their binding affinity towards the PPARα and -γ proteins. The binding affinity values calculated for CGA interactions with both PPAR proteins were over 10-fold higher compared to that of CA, suggesting that for combinatorial treatment with concentrations of CA lower than 50 μM, the predominant activity is due to CGA. Furthermore, dose-dependent interactions between CA and CGA that need additional clarification should be taken into consideration as another possible interpretation of the observed changes in the adipogenic protein levels, especially the PPAR. 

Data from the molecular docking studies also suggested that both phenolic acids express low affinity towards the protein structure of C/EBPα. Therefore, we could speculate that the changes in gene expression and protein abundance upon CA and CGA treatments are a consequence from PPAR modulation in adipocytes rather than a direct pharmacological interaction with C/EBPα. 

## 4. Materials and Methods 

### 4.1. Materials

Buffers and chemicals used to perform electrophoresis, immunoblotting and RT-qPCR were obtained from Bio-Rad Laboratories, Inc. (Hercules, CA, USA). The following antibodies were used for the Western blotting analysis: rabbit anti-C/EBPα (#2295S) and anti-PPARγ antibodies (#2443S) from Cell Signaling Technology (Leiden, The Netherlands); rabbit anti-adiponectin antibody (#GTX112777) from GeneTex Inc. (Ivrine, CA, USA); hFAB rhodamine anti-tubulin (#12004166), hFAB rhodamine anti-glyceraldehyde 3-phosphate dehydrogenase (GAPDH; #12004168) and goat anti-rabbit IgG StarBright Blue 700 (#12004162) antibodies from Bio-Rad. All other materials and analytical-grade substances were purchased from Merck KGaA (Darmstadt, Germany) unless otherwise specified.

### 4.2. In Silico Molecular Docking

Docking calculations were conducted through the Autodock Vina of PyRx 0.8 software. The crystal structures of the target proteins were derived from Protein Data Bank (PDB, www.wwpdb.org) with PDB IDs as follows: 4CFF for AMPK, 1NWQ for C/EBPα; 2P54 for PPARα and 2P4Y for PPARγ. In order to prepare the proteins for the docking simulation, all the water molecules and the co-crystalized heteromolecules were removed, followed by addition of hydrogen atoms and neutralization using Kollman united-atom charges. The dimensions of the grid box were 60 × 60 × 60 with 0.375 Å distance between points. Autodock4 and Lamarckian genetic algorithms were used to dock 250 conformations for each test compound (Molinspiration Database, www.molinspiration.com). Discovery studio 2020 Visualizer was employed to investigate the protein–ligand non-bonding interactions.

### 4.3. Cell Culture and Treatments

Human SGBS preadipocytes were grown in Dulbecco’s Modified Eagle’s Medium/Nutrient F-12 Ham medium enriched with 10% fetal bovine serum, 1% penicillin/streptomycin (10,000 IU/10 mg/mL) solution, 33 μM biotin and 17 μM pantothenic acid. Confluent preadipocytes were subjected to white adipogenic differentiation in serum-free growth media supplemented with 2 μM rosiglitazone, 10 μg/mL apo-transferrin, 20 nM insulin, 25 nM dexamethasone, 500 µM 3-isobutyl-1-methylxantine, 100 nM cortisol and 200 pM triiodothyronine. From the 4th day until the end of the study, the 3-isobutyl-1-methylxantine, rosiglitazone and dexamethasone were omitted from the differentiation medium. 

Stock solutions of ORST, CA and CGA in DMSO were prepared and further diluted in the culture medium. Cells were grouped as follows: non-differentiated SGBS preadipocytes (ND), control differentiated SGBS adipocytes treated with vehicle solution (Vehicle), a positive control group stimulated with ORST 5 μM and nine experimental groups exposed to the respective CA, CGA and CA/CGA concentrations (5, 10 and 50 μM). Treatment concentrations were selected upon cell viability evaluation in SGBS cells at both pre- and post-differentiation states (Supplementary Materials Figure S1). Stimulation was started upon initiation of differentiation and was repeated with every medium renewal for nine days. On the 9th day of differentiation, total RNA and whole-cell protein lysates were extracted, and an ORO assay and free glycerol release measurements were performed. Each assay was carried out at least in triplicate in three independent experiments.

### 4.4. Adipogenesis Evaluation

Following fixation with 10% formalin for 10 min at room temperature (RT), the cells were stained with a fresh filtered ORO dye solution for 15 min at RT. The stained cells were observed and imaged with an Oxion Inverso OX.2053-PLPH microscope and a DC.10000-Pro CMEX camera (Euromex, Arnhem, The Netherlands). ImageFocus Alpha software v1.37.13814 (Euromex) was used for image evaluation.

Total lipid content was assessed by isopropanol extraction of the ORO dye from the cells and subsequent absorbance acquisition at 495 nm on an Anthos Zenyth 340 multiplate reader (Biochrome, Berlin, Germany). 

### 4.5. Adipocyte Lipolysis Quantification

Evaluation of basal lipolysis was performed in cell culture supernatant using the Adipolysis assay kit (#MAK313) from Merck KGaA, following the manufacturer’s protocol. Glycerol standard dilutions of 10 μL or culture medium sample were added to 100 μL working reagent in a 96-well plate. Following incubation for 20 min at RT, the optical density was measured at 570 nm. Glycerol concentration (μM) was calculated according to the obtained standard curve.

### 4.6. RT-qPCR

The Quick-RNA Miniprep kit (#R1055) from Zymo Research (Irvine, CA, USA) was used for total RNA extraction. Reverse transcription to obtain copy DNA templates was performed with the RevertAid First Strand cDNA synthesis kit (#1622) from Thermo Fisher Scientific Inc. (Waltham, MA, USA). Gene expression was quantified by RT-qPCR on a C1000 Touch thermal cycler with a CFX96 detection system (Bio-Rad) using SsoFast EvaGreen Supermix (#1725204). Normalization was done to the expression of *RPL13A* and *TUBB* as reference genes using the comparative threshold cycle (ΔΔCt) method. Primer pairs for the RT-qPCR were used as listed in Supplementary Materials Table S1. 

### 4.7. Western Blot

Cell lysis radioimmunoprecipitation assay (RIPA) buffer freshly supplemented with 1% protease and phosphatase inhibitor cocktail was used for total protein extraction. Total protein content of the collected samples was determined via Bradford assay. Proteins (50 μg per slot) were resolved by SDS-PAGE and transferred to polyvinylidene fluoride membranes with the Trans-Blot Turbo transfer system (Bio-Rad). Membranes were blocked with 5% (*w*/*v*) bovine serum albumin in Tris-buffered saline for 1 h at RT. Incubation with primary antibodies against adiponectin, C/EBPα or PPARγ was done overnight at 4 °C. The StarBright Blue 700 secondary fluorescent antibody was used for detection of the target proteins, followed by incubation with direct antibodies against either tubulin or GAPDH as housekeeping proteins. Blots were visualized with the ChemiDoc MP imaging system (Bio-Rad) and quantified with Image Lab software (Bio-Rad). 

### 4.8. Statistical Analysis

Data evaluation was performed with SigmaPlot software v11.0 (Systat Software GmbH, Erkrath, Germany) and data are presented as mean ± SEM. Comparison between groups was determined by one-way analysis of variance (ANOVA), followed by Tukey’s post hoc test to measure significance between means. Values of *p* < 0.05 were defined as the threshold for significance. 

## 5. Conclusions

Collectively, the obtained data provide evidence that CA/CGA co-treatment promotes anti-obesity effect, partly through the activation of browning of white adipocytes and lipolysis enhancement. While both compounds are known AMPK activators, their potential synergistic effect with respect to obesity has not been adequately explored. Moreover, the effect of the two phenolic compounds as a co-treatment in human adipocytes has not been described previously. Mechanistically, our data reveal that the CA/CGA effect is mediated via AMPK and PPARα/-γ signaling pathways. Interestingly, dual agonistic–antagonistic PPARγ modulation was observed upon CA/CGA co-stimulation in SGBS adipocytes, which warrants further investigation. Thus, our findings open a new perspective that co-treatment with CA and CGA could positively interfere with human adipocyte function and metabolism. Further studies are warranted on the characterization of this combinatorial treatment in the context of in vivo models of obesity and potential implementations into anti-obesity management regimens.

## Supplementary Materials

The following are available online at https://www.mdpi.com/1422-0067/21/24/9740/s1.
