# Supplementary material for: Caffeic and Chlorogenic Acids Synergistically Activate Browning Program in Human Adipocytes: Implications of AMPK- and PPAR-Mediated Pathways
Source: Int J Mol Sci. 2020 Dec 21;21(24):9740. doi: 10.3390/ijms21249740 (PMC7766967; doi:10.3390/ijms21249740)
Supplement: Supplementary file 1 [file ijms-21-09740-s001.pdf]

Supplementary material

Caffeic and Chlorogenic Acids Synergistically Activate Browning Program in Human Adipocytes: Implications of AMPK and PPAR-mediated Pathways

Liliya V. Vasileva [1], Martina S. Savova [1,2], Kristiana M. Amirova [1,2], Zhivka P. Balcheva-Sivenova [1,2], Claudio Ferrante [3], Giustino Orlando [3], Martin Wabitsch [4] and Milen I. Georgiev [1,2,*]

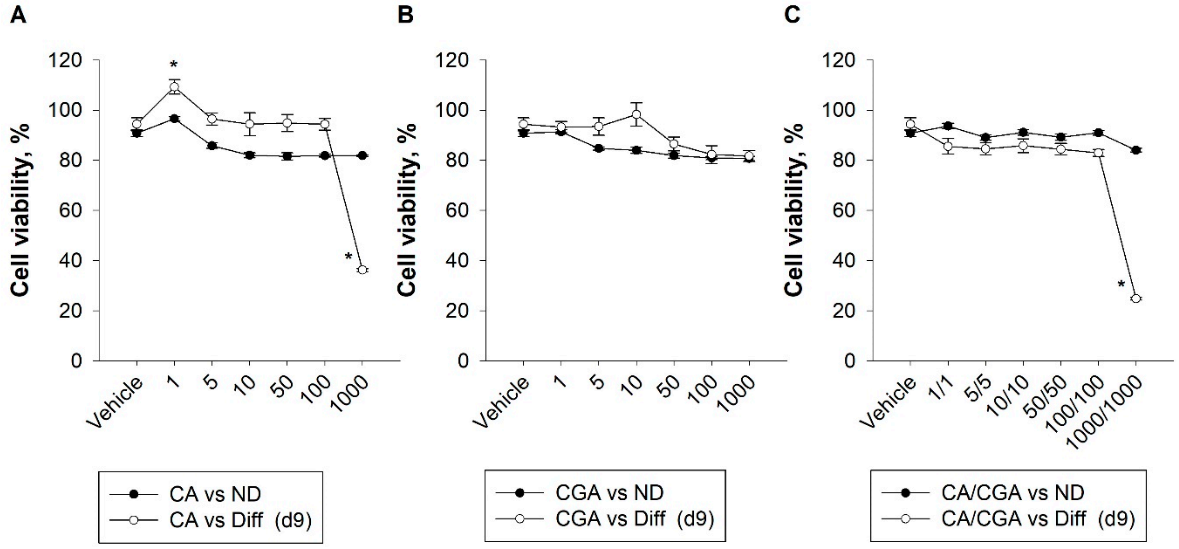

Supplementary Figure S1. Caffeic and chlorogenic acids effect on cell viability of SGBS cells. Caffeic acid (CA, **A),** chlorogenic acid (CGA, **B**) or CA/CGA combination (**C**) in concentrations up to 100 μM do not influence cell viability of both SGBS preadipocytes (ND) and differentiating adipocytes on day 9 (Diff) on MTT assay. Shortly, SGBS cells (5000 cells/well/100 μL in 96-well plates) were grown to near confluence for 48h or differentiated to adipocytes for eight days and treated with increasing concentrations from 1 μM to 1000 μM of CA, CGA, CA/CGA co-treatment or vehicle (0.02% DMSO). Twenty four hours post-treatment 5 mg/mL MTT solution was added (10 μL/well) for 4 h followed by acquisition of the absorbance at 570 nm with reference filter 620 nm. Cell viability is presented in % of vehicle-treated controls mean ± SEM and are representative from three independent experiments.

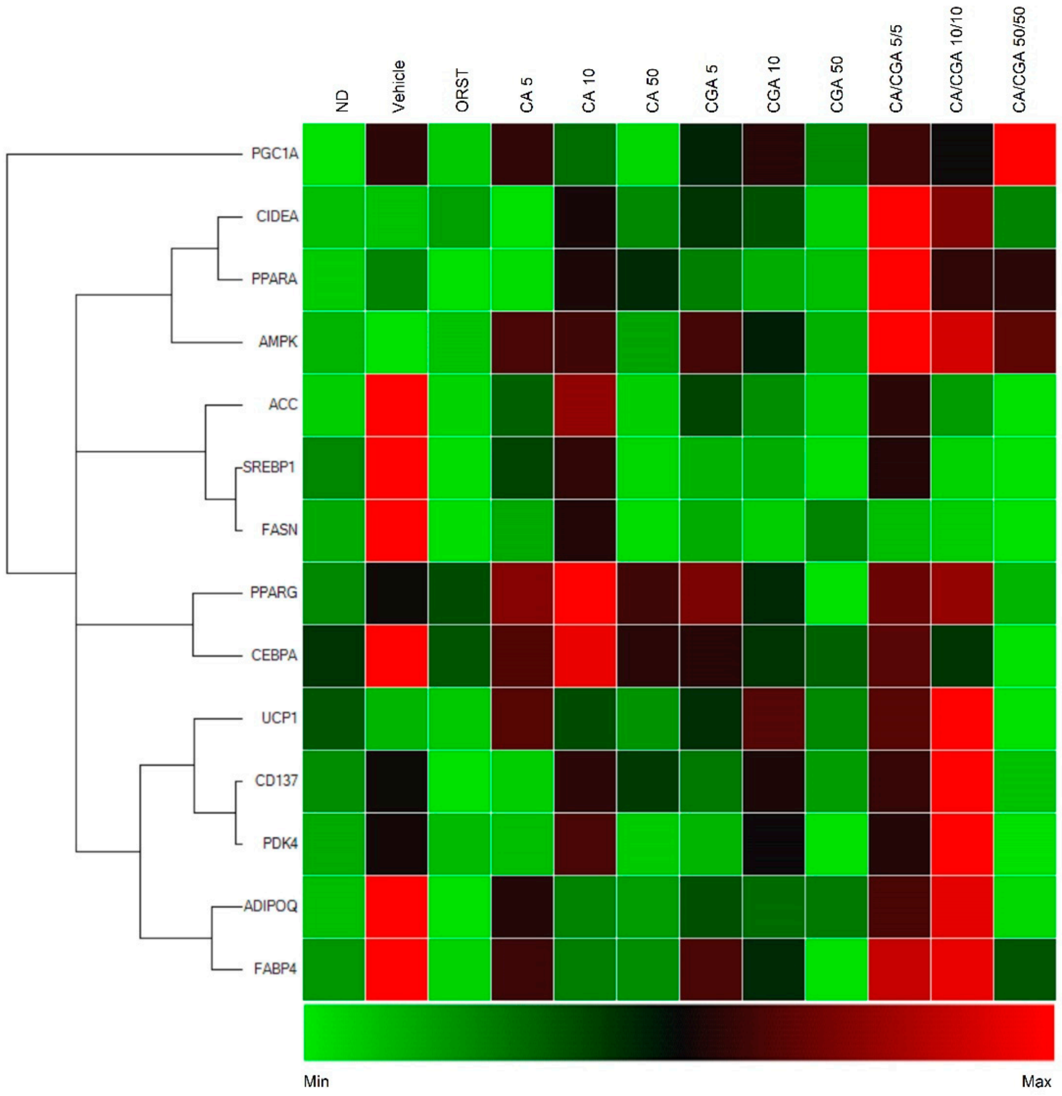

Supplementary Figure S2. Hierarchical cluster analysis and heatmap of the normalized relative expressions from the RT-qPCR. Caffeic and chlorogenic acids co-stimulation induced mRNA expression profile changes resembling browning in human white adipocytes. *RPL13A* and *TUBB* were used to normalize the data. Each sample was analysed in triplicates from three independent experiments. Data are presented in mean ± SEM. *P<0.05 compared to vehicle control group.

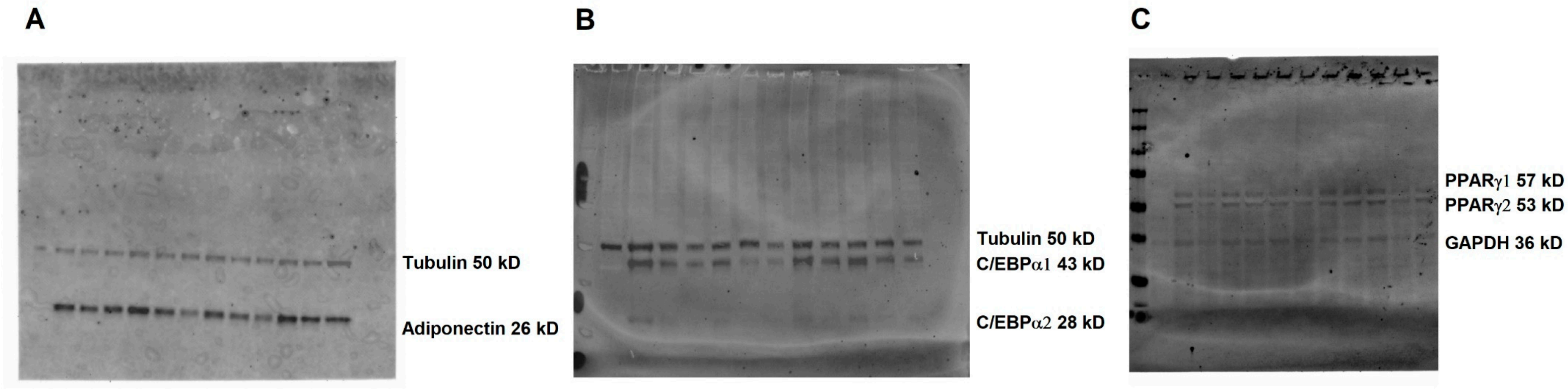

Supplementary Figure S3. Original images of the Western blot analysis from which Figure 5 was prepared. Protein expression levels of C/EBPα (**A**) and adiponectin (**B**) were normalized over tubulin as an internal control while PPARγ was normalized over GAPHD (**C**). Images were acquired on ChemiDoc MP (Bio-Rad) under multichannel mode with fluorescent detection. Lanes were loaded with 50 µg total cell protein lysates as follows: non-differentiated SGBS; Vehicle-treated SGBS adipocytes; orlistat 5 µM, CA 5 µM, CA 10 µM, CA 50 µM, CGA 5 µM, CGA 10 µM, CGA 50 µM, CA/CGA 5/5 µM, CA/CGA 10/10 µM, CA/CGA 50/50 µM. These blots are representative from three independent experiments.

Supplementary Table S1. Primer pairs designed for the RT-qPCR.

Target gene (human)	Forward primer (5' - 3')	Length	Reverse primer (5' - 3')	Length	Tm f/r (°C)	Product length (bp)
ACC	TTCACTCCACCTTGTCAGCG	20	GTCAGAGAAGCAGCCCATCA	20	60.25/59.75	99
ADIPOQ	TGCCCAAAGAGGAGAGAGGAA	21	TCAGAAACAGGCACACAACTCA	22	60.49/60.36	97
AMPK	GAAAGTCGGCGTCTGTTCCA	20	CATGTGTGCATCAAGCAGGA	20	60,60/58.83	111
CD137	AATGGGACGAAGGAGAGGGA	20	AGAAACGGAGCGTGAGGAAG	20	59.96/60.04	187
CEBPA	TATAGGCTGGGCTTCCCCTT	20	CTAGGTCTCCCTCTCCCACC	20	60.03/60.11	148
CIDEA	CAGCAAGACTCTGGATGCCC	20	CAAGATCATGAAATGCGTGTTGTCT	25	60.75/60.62	130
FABP4	ACCTTAGATGGGGGTGTCCT	20	TGCGAACTTCAGTCCAGGTC	20	59.58/59.97	177
FASN	TCTACGGCTCCACGCTCTT	19	GAAGAGTCTTCGTCAGCCAGG	21	60.68/60.40	130
PDK4	CCTGTGAGACTCGCCAACAT	20	GCTTTCTGGTCATCTGGGCT	20	60.04/60.03	152
PGC1A	AAATATCTGACCACAAACGATGACC	25	GTTGGTTTGGCTTGTAAGTGTTGT	24	59.65/60.62	134
PPARA	CGGGATGCTGGTAGCGTATG	20	GCCAGGACGATCTCCACAG	19	60.67/59.86	187
PPARG	GATCCAGTGGTTGCAGATTACAA	23	GAGGGAGTTGGAAGGCTCTTC	21	58.99/60.07	144
RPL13A	AAAAGCGGATGGTGGTTCCT	20	GCTGTCACTGCCTGGTACTT	20	59.89/59.96	118
SREBP1	TGTACTTCTGGAGGCATCGC	20	CTACAAGCCAGGTCCAGGTG	20	59.82/60.04	139
TUBB	AGCCGTCTTACTCAACTGCC	20	GTCACCCAGAATGGCAGAA	19	60.04/59.96	198
UCP1	GAAACAGCACCTAGTTTAGGAAGC	24	AGCCTTCGGTTGTTGCTATTATTCT	25	59.85/60.86	187

Abbreviations: bp, base pairs; Tm, melting temperature.
